# Increased Antibiotic Resistance in Children with *Helicobacter pylori* Infection: A Retrospective Study

**DOI:** 10.3390/pathogens11020178

**Published:** 2022-01-27

**Authors:** Luca Helmbold, Beniam Ghebremedhin, Aliyah Bellm, Marc A. Hopkins, Stefan Wirth, Malik Aydin

**Affiliations:** 1 Center for Child and Adolescent Medicine, Center for Clinical and Translational Research (CCTR), Helios University Hospital Wuppertal, Witten/Herdecke University, 42283 Wuppertal, Germany; luca.helmbold@uni-wh.de (L.H.); marc.hopkins@helios-gesundheit.de (M.A.H.); 2 Institute of Medical Laboratory Diagnostics, Center for Clinical and Translational Research (CCTR), Helios University Hospital Wuppertal, Witten/Herdecke University, 42283 Wuppertal, Germany; Beniam.ghebremedhin@helios-gesundheit.de; 3 Helios Hospital Krefeld, Children’s Hospital, Teaching Hospital of RTWH University Hospital Aachen, 47805 Krefeld, Germany; aliyah.bellm@helios-gesundheit.de; 4 Laboratory of Experimental Pediatric Pneumology and Allergology, Center for Biomedical Education and Research, School of Life Sciences (ZBAF), Faculty of Health, Witten/Herdecke University, 58455 Witten, Germany

**Keywords:** abdominal pain, children, multiple drug resistance, antibiotic resistance, histopathology, gastritis, *Helicobacter pylori*

## Abstract

Children with recurrent abdominal pain may be suffering from a *Helicobacter* *pylori* (HP) infection. The gold standard for confirming HP gastritis is histological evaluation and microbiological tests performed on specimens collected by esophagogastroduodenoscopy (EGD). The aim of this study was to analyze HP positive cultures and antibiograms with regard to clinical and histopathological correlates. The data of 124 subjects with frequent gastrointestinal symptoms who underwent an EGD were retrospectively collected and analyzed. The mean age of the patients was 13 ± 3.6 years. The most frequent complaints were epigastric pain (84%; *n* = 100/119) and dyspepsia (79%; *n* = 94/119). HP gastritis was diagnosed in 54% (*n* = 67). Interestingly, 40% (*n* = 49) of the isolates were resistant to at least one antibiotic: amoxicillin (20%; *n* = 10/49), clarithromycin (45%; *n* = 22/49), or metronidazole (59%; *n* = 29/49). Isolates were resistant to two or more antibiotics in 16% (*n* = 20) of cases. In conclusion, we revealed remarkably high resistance rates to amoxicillin, metronidazole, and clarithromycin in our cohort. The presence of antibiotic resistance to more than one antibiotic was substantially increased in our HP-infected patients and this may negatively affect eradication treatment.

## 1. Introduction

In clinical practice, pediatric patients with recurrent or chronic abdominal pain (CAP) and suspected *Helicobacter pylori* (HP) infection pose a well-known challenge for physicians [1]. In this context, recurrent abdominal pain (RAP) is defined as chronic intermittent pain episodes associated with functional impairments for more than three months in a course [2,3]. Most studies in children do not show any correlation between the described symptoms and HP infection [4]. Nevertheless, HP infection with associated gastritis is occasionally linked to epigastric abdominal pain [4,5]. Additional symptoms may include, e.g., dyspepsia, heartburn, nausea, emesis, constipation, or diarrhea [4]. In general, the prevalence of HP infections can also be associated with socioeconomic status [6]. Immigrant children living in Germany were 21% (28%) [7,8] more likely to be diagnosed with HP than children born in Germany (7%) [8]. The current requirements for diagnosis in pediatric patients are a positive HP culture or a conspicuous histopathology with one other positive invasive test result [1]. Thus, an esophagogastroduodenoscopy (EGD) with histopathological biopsy is the recommended procedure to confirm the diagnosis of HP gastritis [9]. However, non-invasive tests such as the HP antigen stool test or ^13^C-urea breath tests are still frequently performed in the post-inpatient outcome monitoring setting [10]. The HP antigen stool test seems more reliable than the ^13^C-urea breath test in children under the age of six years due to patient compliance and test accuracy [11,12]. A widely accepted recommendation for eradication therapy consists of amoxicillin combined with either clarithromycin or metronidazole, and a proton-pump inhibitor [1]. Over the last decade, the resistance rates of HP strains to these antibiotics have been alarmingly increasing in childhood [13].

Considering this important observation, we performed a retrospective, single-center study to analyze the correlation between clinical symptoms, HP infection, and the results of laboratory findings. The aim was to explore the antibiotic resistance rates at our center, which serves a heterogenic patient population with diverse ethnic and socioeconomic backgrounds.

## 2. Materials and Methods

Data from 124 children (≥1 to <17 years of age) with suspected HP gastritis who underwent an EGD between October 2015 and July 2019 at a tertiary pediatric center of the Helios University Hospital Wuppertal, Witten/Herdecke University, Wuppertal, Germany, were retrospectively analyzed. Patient records were included only once during the study period. The inclusion criteria were epigastric and/or abdominal pain, nausea, emesis, diarrhea, heartburn, positive HP tests, and a prior diagnosis of gastritis. Subjects with suspected inflammatory bowel or celiac disease were excluded. As per the current guidelines from The European Society for Paediatric Gastroenterology Hepatology and Nutrition (ESPGHAN) and the North American Society for Pediatric Gastroenterology, Hepatology & Nutrition (NASPGHAN), HP testing was performed after stopping the proton-pump inhibitors (PPI) for 2 weeks and antibiotic use for 4 weeks before the EGD.

All EGDs were performed by the same pediatric gastroenterologist using EG29-i10 and EG-2790K endoscopes by PENTAX Medical Europe GmbH, Hamburg, Germany, reducing the risk of inter-operator bias. For HP testing, at least two gastric biopsies were taken for histopathological and microbiological analyses. A polymerase chain reaction (PCR) was not performed. The patient cohort was stratified by sex, age, age- and sex-specific body mass index (BMI) percentile, symptoms, medical history (RAP, eradication treatment, and PPI intake), macroscopic imaging (gastric antrum and corpus), and histopathological and microbiological status (rapid urease test (RUT), culture, and antibiogram). Our study was based on the ethical standards of the Declaration of Helsinki from 1964 and its later amendments. Ethics approval was obtained from the Ethics Committee of the Witten/Herdecke University, Germany (166/2019). The data extraction and analyses were performed retrospectively and pseudonymously; participant informed consent was not necessary. The biostatistical analyses were performed using Microsoft^®^ Office 365 Excel for Mac (Version 16.35) and IBM^®^ SPSS^®^ Statistics (Version 26). The descriptive data are presented by using absolute and relative numbers as well as mean, median, and standard deviation. The *p*-value was set at *p* < 0.05. Cross tables were used to bivariately determine the relative frequency of possible factors (BMI, symptoms, PPI intake, non-invasive tests etc.) and a positive HP culture, histopathology, or antibiotic resistance. We then used the Pearson chi-square test to prove significant correlation between the variables. The power of this correlation was then measured using Phi, Cramer’s V, or the contingency coefficient (CC).

### 2.1. H. pylori Culture Conditions

The biopsy specimens were placed in a transport medium. The specimens were seeded on Becton Dickinson (BD) *Helicobacter pylori* selective agar plates (Heidelberg). The BD *Helicobacter pylori* selective agar consisted of Columbia agar, including, e.g., vancomycin and amphotericin for growth inhibition of contaminating flora. The plates were subsequently stored in a multi-gas incubator (microaerophilic atmosphere: 10% carbon dioxide, 5% oxygen, and 85% nitrogen at 37 °C) for 3 to 7 days. If HP was not isolated after 7 days of incubation, the plates were incubated for another three days. HP isolates were identified based on colony morphology and were confirmed by matrix-assisted laser desorption/ionization time-of-fight mass spectrometry (MALDI-TOF) using a Microflex LT system (Bruker Daltonics, Bremen, Germany). The measured profiles were compared to a profile hosted on a database using MALDI Biotyper 3.1 software (Bruker Daltonics). The colonies were confirmed by subculture analysis. Plates containing Mueller–Hinton agar (MHA) plus 10% horse blood were inoculated with a swab from the suspension at a 0.5 McFarland standard. Antimicrobial disks (10 µg amoxicillin, 15 µg clarithromycin, and 5 µg metronidazole; Besbion, Liofilchem MTS, Roseto degli Abruzzi, Italy) were placed on the same plate for each HP isolate. The Mueller–Hinton (supplemented with horse blood) agar plates were incubated for 72 h at 37 °C in a microaerophilic atmosphere. Reading of the agar dilution method (ADM) was performed after 96 h incubation period. The inhibition zone diameter was measured in millimeters using a ruler: susceptibility was assumed for amoxicillin at ≥25 mm, for clarithromycin at ≥21 mm, and for metronidazole at ≥21 mm inhibition zone diameters [14].

### 2.2. H. pylori Antimicrobial Susceptibility Testing (AST)

The antimicrobial susceptibility testing (AST) was performed using minimum inhibitory concentration (MIC) test strips for five antibiotics: amoxicillin, clarithromycin (MIC range was 0.016 to 0.125), metronidazole (64 to 256 μg/mL), tetracycline, rifampicin, and levofloxacin (Liofilchem MTS, Roseto degli Abruzzi, Italy), which are all frequently used in HP eradication protocols. Susceptibility to these antibiotics was determined by inoculating 0.5 MacFarland on Mueller–Hinton (supplemented with horse blood) agar and placing an E-test strip on the agar afterward. This was incubated at 37 °C for three days under micro-ventilation conditions (microaerophilic), and the presence of bacterial colonies was observed by the E-test. The in vitro MICs of the five antibiotics for the HP clinical isolates were defined by the points of intersection of the inhibitory zones with the strips.

To determine the susceptibility, we adopted the European Committee on Antimicrobial Susceptibility Testing (EUCAST) guidelines (www.eucast.org; accessed on 7 July 2021). The MIC breakpoints for amoxicillin, clarithromycin, metronidazole, tetracycline, rifampicin, and levofloxacin are >0.125, >0.5, >8, >1, >1, and >1 mg/L, respectively. For quality control, HP strain ATCC 43504 was selected. Resistance to two or more antimicrobials was defined as multidrug resistance (MDR) [15]. Subjects showing resistance to two or more classes of antimicrobial agents were regarded as having multidrug resistance.

## 3. Results

In the following results, our primary aim was to evaluate the data with regard to histological and microbiological characteristics. In addition, we analyzed the observed single and multidrug resistance rates.

### 3.1. Patient Characteristics

In total, 124 patients were retrospectively analyzed (Table 1). Data showed that 35% (*n* = 43) of the study subjects were boys with a median age of 13 years (range = 15 years) and 65% (*n* = 81) were girls with a median age of 14 years (range = 16 years). The majority of the study population (*n* = 71; 57%) demonstrated a BMI between P26 and P90 (percentile curves). The BMI was, on average, 20.6 ± 6.0 kg/m^2^. Of all patients, 96% (*n* = 119) were 6 years of age or older and were able to express specific complaints. The most frequent symptoms of these 119 patients were epigastric pain (*n* = 100; 84%), dyspepsia (*n* = 94; 79%), and nausea (*n* = 20; 17%). Furthermore, 26% (*n* = 31) reported recurrent abdominal pain in their medical history. Of the 124 patients, 17 (14%) had previously received an eradication treatment for HP. In addition, 23 (19%) were on long-term PPI treatment. The parents’ countries of origin were unknown. In prior outpatient care, 27% (*n* = 34) of the 124 patients had a positive ^13^C-urea breath test and 29% (*n* = 36) had a positive HP stool antigen test.

### 3.2. Histopathological and Microbiological Findings

A typical endoscopic finding, such as nodular gastric mucosal hyperplasia, was mostly detected in the gastric antrum (*n* = 93; 75%) and corpus (*n* = 43; 35%). As a result of the histopathology and further biopsy-based tests (culture and RUT), 54% (*n* = 67) of the patients had been diagnosed with HP gastritis. Furthermore, 94 (76%) of the 124 individuals had conspicuous histopathology, 92 (74% of the 124) of whom showed valid test results in the microbial cultivation.

In 39 (31%) of the 124 cases, we found a positive RUT. Positive histopathology was a distinct indicator for a positive RUT (chi-square (1) = 14.72, *p* < 0.001, phi/Cohen’s w = 0.374) (Supplementary Materials Table S1). Overall, only nine (7%) patients had positive histopathology and a RUT without a positive culture result.

Beyond that, HP was isolated in the cultures of 58 (47%) patients. One case had normal histology but a positive HP culture. In 62% (*n* = 36) of cases, the subjects showed BMI with percentiles between P26 to P90, and the median age was 13 years (range = 16 years). There was no association between a positive culture and reported symptoms (epigastric pain, dyspepsia, and nausea; *p* > 0.05) or former eradication therapy (*n* = 7; 12%; *p* = 0.501). Moreover, we did not find any correlation between the successful cultivation of HP and specific BMI percentile curves (*p* = 0.820). Fewer positive HP cultures (*n* = 5; 25%) were found in cases with reported previous PPI intake (*n* = 20).

Only 23 of 58 patients with a positive HP culture presented a positive ^13^C-urea breath or a HP stool antigen test before intervention. Unfortunately, there were no data available to us on many non-invasive test results (^13^C-urea breath (*n* = 29/58) and the stool antigen test (*n* = 34/58)). The missing data were presumably collected in primary outpatient care and were not documented during the following hospital admission. The RUT was positive in 27 (47%) and negative in 19 (33%) of 58 cases with successful cultivation of HP. The RUT results of the remaining 12 positive cultures were not documented and are unknown.

Finally, in 43 (35%) of the 124 cases, the results of RUT and culture were negative, and a HP infection could not be detected. Following this, 56% (*n* = 24) of the uninfected cases belonged to patients with BMI percentile curves between P51 to P97, and with a median age of 15 years (range = 13 years). Of the enrolled patients, 35 (81%) and 37 (86%) reported epigastric pain and dyspepsia, respectively. The rate of previous eradication treatment or PPI intake was negligible.

### 3.3. Antibiotic Resistance

We tested 58 HP isolates for antimicrobial resistance to six different antibiotics: amoxicillin, clarithromycin, metronidazole, tetracycline, rifampicin, and ciprofloxacin. In 51 HP isolates, an antibiogram was suspicious for resistance to at least one antibiotic, specifically to amoxicillin, clarithromycin, or metronidazole in 49 HP isolates. Seven isolates did not have a documented antibiogram or one was not performed. All above-mentioned samples were taken from subjects in whom HP gastritis was confirmed, and complaints such as epigastric pain, dyspepsia, or recurrent abdominal pain were noted in the history of present illness. Of these common antibiotics, high single resistance rates were found for amoxicillin (20%; *n* = 10/49), metronidazole (59%; *n* = 29/49), and clarithromycin (45%; *n* = 22/49). The resistance rates for tetracycline were 12% (*n* = 6/51), 31% for ciprofloxacin (*n* = 16/51), and 22% for rifampicin (*n* = 11/51).

The highest frequency of co-resistance was found for clarithromycin and metronidazole (22%; *n* = 11/49). In addition, the frequency of co-resistance against amoxicillin plus clarithromycin or metronidazole or triple antibiotic resistance was 6% (*n* = 3/49) in each combination (Table 2). We could not find any correlation between resistance rates and age or BMI. Moreover, there were no differences in antimicrobial resistance to specific antibiotics over the study period.

We did not consider previous diagnostics, e.g., the ^13^C-urea breath test or the stool antigen test, due to limited data. Furthermore, subjects who presented several times during the study period were not included multiple times with regard to changes in histopathology or antibiotic resistance. The majority (*n* = 43/51) of enrolled patients had received their first eradication treatment and showed higher HP resistance rates. Only in seven of the remaining eight cases, a previous eradication therapy was clearly known in the medical history, and secondary or tertiary HP resistance could be assumed. In these seven patients, two were resistant to amoxicillin, one to metronidazole, and four to clarithromycin. In our cohort of 124 enrolled patients, we did not statistically (Pearson chi-squaretest) detect any significant correlation between a prior known eradication therapy and a specific antibiotic resistance. Nevertheless, a very high amount of resistance and relevant co-resistance to common antibiotics were identified.

In a small group, we additionally performed an agar diffusion assay to compare the effectiveness of E-test and ADM. In comparison of the two methods for amoxicillin, the susceptibility results were discrepant in only one case (subject 1) and for clarithromycin in another case (subject 4). However, apart from these discrepancies, the two antimicrobial susceptibility methods revealed equivalent results for all tested antimicrobials in HP (Supplementary Materials Table S2).

## 4. Discussion

Overall, we analyzed children and adolescents with abdominal complaints to reveal potential single and multidrug HP resistance. Today, the required goal for HP treatment is an eradication rate of 90% to avoid additional antibiotic use [16]. Lower antimicrobial susceptibility to antibiotics and higher risk of treatment failure remain significant challenges [17]. In practice, eradication rates are around 70%, as the described 90% can be rarely achieved even under study conditions [18]. Jones et al. [1] recommended that the diagnosis of HP infection be mainly based on a positive culture or positive HP gastritis by histopathology and biopsy-based tests. Antimicrobial susceptibility testing is also highly recommended [1]. In this manner, the majority of our study population was diagnosed by positive HP culture with following antimicrobial susceptibility testing.

We found remarkable elevated single and multi-drug resistance rates in our cohort, especially to amoxicillin (10/49; 20%). The resistance rates to clarithromycin (22/49; 45%) and metronidazole (29/49; 59%) were far above the recommended limits of 15% and 20%, respectively, for use as first-line agents [1,19], and were higher than other published European data [17,20,21]. In the USA, there are comparable resistance rates to clarithromycin (32%), which are even higher in cases with previous clarithromycin-containing therapies [22]. Biernat et al. [20] reported in a comparable study in Poland, a total resistance rate of 37% for metronidazole. In another relevant study, the resistance to metronidazole was only 3% [23].

Kori et al. [17] analyzed data from 165 mostly southern European children with previous HP infection and a failed treatment. In this cohort, they revealed an exceptionally high resistance rate to metronidazole (52.4%). The resistance to metronidazole from antibiotic-naïve patients in our study was even higher (59%). Khoury et al. [24] and Talebi Bezmin Abadi et al. [25] both described resistance to metronidazole with rates of over 60% in Israeli and Iranian patients. Our results might reflect the larger proportion of immigrants from southern Europe, Africa, and the Middle East in our cohort [17]. Other causes of such high rates of resistance remain unclear and should be a part of future studies. In this sense, eradication therapy with metronidazole would not be recommended to the patient population in this study [19]. In addition, Kotilea et al. [10] described increasing resistance rates to quinolones and amoxicillin. The resistance rate to ciprofloxacin in our study corresponds with their work. Strikingly, almost one in five patients in our cohort with a conspicuous antibiogram revealed antimicrobial resistance to amoxicillin. In comparison to Regnath et al. [13] and Kori et al. [17], our amoxicillin resistance rate was alarmingly high and seems to be an example of regional variability in antibiotic resistance rates. In contrast to the tendency presented above, Bluemel et al. [26] and Hofreuter et al. [27] found an acceptable resistance rate to clarithromycin, which was below the recommended threshold.

In our laboratory, we barely reached the 3.0 MacFarland standard for the E-test, so we performed AST with a 0.5 MacFarland standard. However, reading the E-test strips after 72 h was possible without any significant issues. In Indonesia, HP isolates were tested via E-test versus ADM [28]. Among the 72 isolates tested, the E-test’s results showed a higher prevalence of resistance to all the antibiotics tested but the difference was not significant. The results provided increased essential agreement between the two methods (>90.0%) for all the antibiotics tested, but only 84.7% for metronidazole [28]. The authors concluded, even though some discrepancies were observed, that the E-test has acceptable agreement for levofloxacin, metronidazole, tetracycline, and clarithromycin but further confirmation may be necessary for amoxicillin [28].

In general, the E-test may overestimate the rates of resistance to antibiotics. Best et al. [29] suggested that agar dilution is useful in studying large numbers of strains and that the E-test can be used to discover the susceptibility status of strains from individual patients. Moreover, the E-test is considered a good method to determine the MIC of clarithromycin and amoxicillin against HP [29,30,31], with good intra- and inter-laboratorial correlation [32].

The simplest, most cost-effective, and most frequently used method for routine susceptibility testing is the disc-diffusion method. However, it is not recommended for slow-growing microorganisms such as HP due to irregular antibiotic release from the discs. This is in contrast to the E-test, which is useful for slow-growing bacteria [33,34]. HP strains at a density of 2.0 McFarland standard yielded approximately 10^8^ CFU/mL. Agar dilution plates were inoculated with 5 μL, or approximately 5 × 10^5^ CFU, and plates with E-test strips were inoculated with 100 μL, or approximately 1 × 10^7^ CFU [29,32,35,36]. In conclusion, the E-test method presented better agreement with the gold standard of diagnosis.

There are several molecular aspects that may be associated with increased antibiotic resistance. Next-generation sequencing (NGS) technology will be an important tool to analyze the metagenome of HP, which may hold clues to the causes of antibiotic resistance [37]. Future studies should include a more precise histopathological determination of the genotype variants of HP in this cohort to detect multi-strain HP infections with antibiotic hetero-resistance [38]. Moreover, antimicrobial resistance rates vary depending on geographic locations on the local, national, and international levels [1]. Thus, regional antibiotic resistance data could improve individual treatment regimens for HP infections [19].

Our research is limited by its retrospective study design and the small cohort number. Other limitations, e.g., missing data or incomplete data recording, were unavoidable. The study was performed at a single center. Furthermore, we could not differentiate resistance rates based on prior HP eradication therapy or other antibiotic treatments due to the limits of statistical analysis and the small percentage of previously treated subjects. The country of birth of the parents was also not considered in our evaluation.

An additional limitation is that multiple HP strains with different antibiotic resistance profiles (heterogeneity) can exist within the same patient [39,40] that can alter potential microbiological results and resistance tests.

Nevertheless, we were able to emphasize the importance of culture and the use of an antibiogram for the diagnosis and treatment of HP. Moreover, we presented first important data regarding high antibiotic resistance rates in our patient population.

Our study is a relevant example of specific regional distribution of antimicrobial susceptibility and complies with current guideline recommendations. The study parameters reflect the common diagnostic pathway and clinical practice for HP infection in children and adolescents.

## 5. Conclusions

The rising prevalence of HP and multiple antibiotic resistances are global challenges that increasingly affect the safety and quality of the European pediatric health care system. The risks of failed eradication treatment and additional unnecessary procedures are alarming. In our study, specific symptoms or prior drug therapies did not influence the rate of HP infection or antibiotic resistance status. The histopathological findings in HP-infected children are generally important and are well-known diagnostic criteria in guidelines, and successful cultivation and antibiotic resistance testing of HP are crucial for the diagnostic pathway as well as for potential eradication treatment, as shown in the literature. Our revealed amoxicillin resistance of HP was exceptionally high, perhaps pointing to an overall increase in amoxicillin resistance in Europe in the near future. Furthermore, regional specific characteristics of antibiotic resistance and the outcome of prescribed regimens must be further evaluated. The knowledge of a local antibiotic resistance rate against amoxicillin and metronidazole offers a better chance of successful treatment. Future research should focus on regional differences and multiple antibiotic resistance of HP strains linked to histopathological findings and genotypes is still required. An increase in data would help with enhancing the eradication rate of HP and minimizing unnecessary additional therapies and risks for children in healthcare.

## Figures and Tables

**Table 1:** Descriptive overview of patient characteristics, clinical and analytical data.

**Positive Items—Patient Cohort** **(*n* = 124)**	
Age *(mean ± SD, years)*	13 ± 3.6
Ratio *(female/male)*	2:1
BMI *(mean ± SD, kg/m^2^)*	20.6 ± 6.0
BMI P26–P90 *(n, %)*	71 (57%)
Eradication treatment *(n, %)*	17 (14%)
PPI-intake *(n, %)*	23 (19%)
^13^C-urea breath test *(n, %)*	34 (27%)
HP stool antigen test *(n, %)*	36 (29%)
**Verbalized Complaints—Patient Cohort ≥ 6 Years of Age** **(*n* = 119)**	
Epigastric pain *(n, %)*	100 (84%)
Dyspepsia *(n, %)*	94 (79%)
Nausea *(n, %)*	20 (17%)
Recurrent abdominal pain *(n, %)*	31 (26%)

**Table 2:** Single and multidrug resistance of amoxicillin, metronidazole, and clarithromycin. In total, 49 of 51 antibiograms were analyzed for resistance to amoxicillin, metronidazole, and clarithromycin. The very frequent resistance to amoxicillin and metronidazole was particularly striking. In addition, a multidrug resistance rate of 41% (*n* = 20/49) with a proportion of 45% (*n* = 9/20) of amoxicillin-resistant HP isolates is a cause for major concern.

Tested Antibiotics	Frequency	Percentage
Total Amoxicillin	10/49	20%
Amoxicillin	1	
Amoxicillin + Clarithromycin	3	
Amoxicillin + Metronidazole	3	
Amoxicillin + Clarithromycin + Metronidazole	3	
Total Clarithromycin	22/49	45%
Clarithromycin	5	
Clarithromycin + Metronidazole	11	
Clarithromycin + Amoxicillin	3	
Clarithromycin + Amoxicillin + Metronidazole	3	
Total Metronidazole	29/49	59%
Metronidazole	12	
Metronidazole + Clarithromycin	11	
Metronidazole + Amoxicillin	3	
Metronidazole + Amoxicillin + Clarithromycin	3	

## Data Availability

The data can be shared upon reasonable request from the first or senior authorships.

## Supplementary Materials

The following supporting information can be downloaded at: https://www.mdpi.com/article/10.3390/pathogens11020178/s1, Table S1: Statistical tests regarding correlation between rapid urease test (RUT), stool antigen test, and histopathology; Table S2: Comparison of E-test and Agar diffusion test.

## List of Abbreviations

HP: *Helicobacter Pylori*; EGD: esophagogastroduodenoscopy; CAP: chronic abdominal pain; RAP: recurrent abdominal pain; ADM: agar dilution method; AST: antimicrobial susceptibility testing; MIC: minimum inhibitory concentration; MDR: multidrug resistance; BMI: body mass index; PPI: proton pump inhibitor; NGS: next-generation sequencing.
