# Supplementary material for: Increased Antibiotic Resistance in Children with Helicobacter pylori Infection: A Retrospective Study
Source: Pathogens. 2022 Jan 27;11(2):178. doi: 10.3390/pathogens11020178 (PMC8877488; doi:10.3390/pathogens11020178)
Supplement: Supplementary file 1 [file pathogens-11-00178-s001.zip › pathogens-1557218-supplementary.pdf]

Table S1. Statistical tests regarding correlation between rapid urease test (RUT), stool antigen test, and histopathology.

**Stool antigen test – RUT (n = 35)**	***Fisher's exact test*: *p* = 0.119**
Histopathology – RUT (n = 105)	*Pearson chi-square test*: Chi-Square (1) = 14.72, $p < 0.001$, phi/Cohen's w = 0.374

Table S2. Comparison of E-test and Agar diffusion test.

	**subject 1**	**subject 2**	**subject 3**	**subject 4**
**Age [years]**	11	8	15	14
**Chronic symptoms**	yes	yes	yes	yes
**Pre-Helicobacter pylori**	+	+	+	+
**Amoxicillin**				
E-test	susceptible	susceptible	susceptible	susceptible
Agar diffusion test	resistant	susceptible	susceptible	susceptible
**Clarithromycin**				
E-test	resistant	susceptible	susceptible	susceptible
Agar diffusion test	resistant	susceptible	susceptible	resistant
**Metronidazole**				
E-test	resistant	resistant	susceptible	susceptible
Agar diffusion test	resistant	resistant	susceptible	susceptible
